# Breadth versus depth: Cumulative risk model and continuous measure prediction of poor language and reading outcomes at 12

**DOI:** 10.1111/desc.12998

**Published:** 2020-06-22

**Authors:** Marianna E. Hayiou‐Thomas, Emily Smith‐Woolley, Philip S. Dale

**Affiliations:** ^1^ Department of Psychology University of York York UK; ^2^ The Physiological Society London UK; ^3^ Department of Speech & Hearing Sciences University of New Mexico Albuquerque NM USA

**Keywords:** language, outcome, prediction, reading, risk

## Abstract

This study examines whether, and how, multiple risks in early childhood are associated with an increased likelihood of a poor language or literacy outcome in early adolescence. Using data from 210 participants in the longitudinal Twins Early Developmental Study, we focus on the following risk factors at age 4: family risk, and poor language, speech, emergent literacy and nonverbal skills. The outcomes of interest at age 12 are language, reading fluency and reading comprehension. We contrast a ‘cumulative risk’ model, counting the presence or absence of each risk factor (breadth), with a model that also considers the severity of the early deficits (depth). A ‘cumulative risk index’ correlated modestly but significantly with outcome (*r* = 0.32–0.40). Odds ratios confirmed that having many risk factors (3–6) confers a higher probability of a poor outcome (OR 7.86–17.71) than having one or two (OR 3.65–7.28). Logistic regression models showed that predictive validity is not improved by including information about the severity of each deficit. Even with rich information on children's risk status at age 4, we can make only a moderately accurate prediction of the likelihood of a language or literacy disorder 8 years later (Area Under the Curve = 0.74–0.84; Positive Predictive Value = 0.33–0.55, Negative Predictive Value = 0.86–0.91). Taken together, and consistent with the idea of ‘cumulative risk’, these results suggest that the breadth of risk is a core predictor of outcome, and furthermore, that the severity of early deficits does not add significantly to this prediction.

## Graphical abstract

The greater the number of risk factors in early childhood, the greater the likelihood of a poor language or literacy outcome in early adolescence. However, the severity of early deficits may not in itself be very informative: depth (severity) adds little to prediction beyond breadth (number of risk factors).

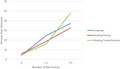

**Research highlights**

1. The greater the number of risk factors in early childhood, the greater the likelihood of a poor language or literacy outcome in early adolescence.
2. The severity of early deficits may not in itself be very informative: depth (severity) adds little to prediction beyond breadth (number of risk factors).
3. Difficulties may emerge in later childhood despite the absence of red flags in the early years.

## 1 INTRODUCTION

There is growing recognition that common neurodevelopmental disorders such as reading disorders and developmental language disorder (DLD) may be the consequence of multiple underlying neurocognitive deficits. This has been explicitly proposed in the ‘multiple deficit’ model of dyslexia (Pennington, 2006; Pennington et al., 2012), and is in line with current thinking in developmental psychology, as well as behavioural and psychiatric genetics, where multiple genetic and environmental risk factors are thought to accumulate, resulting in the behavioural manifestation of a disorder (Evans, Li, & Whipple, 2013). An important corollary of this approach is that the greater the number of risk factors present, the greater the likelihood of a poor outcome: that is, breadth matters. An intuitive, alternative (but not mutually exclusive) possibility is that the severity of the deficits is important in determining the likelihood of a poor outcome: that is, depth matters. In the current paper, we investigate whether the presence of a greater number of risk factors in young children (aged 4) is associated with a greater likelihood of a poor outcome in language and literacy skills at 12. We then consider whether this long‐range prediction is enhanced if the severity of early deficits, beyond just their presence or absence, is taken into account.

The outcomes of interest are oral language difficulties, poor reading fluency and poor reading comprehension at 12. Difficulties in these domains are associated with poor academic performance (Stothard, Snowling, Bishop, Chipchase, & Kaplan, 1998), as well as worse outcomes in broader life domains such as mental health and employment (Law, Rush, Schoon, & Parsons, 2009). The transition to secondary school is challenging for many children, and especially for those with communication and literacy difficulties (Dockrell & Lindsay, 2007). In the current study, we examine the extent to which risk factors identifiable when children first enter formal education (age 4–5) are informative about children's outcomes at the age of transition from primary to secondary school.

### 1.1 Risk factors for poor language and literacy outcomes

It has long been known that both language and literacy disorders run in families, and family aggregation studies clearly show an increased incidence in the relatives of children with dyslexia or language disorder (Flax et al., 2003). A meta‐analysis of prospective studies showed that 45% of children with a first‐degree relative who has dyslexia are likely to develop dyslexia, compared to 11.6% of children who are not at family risk (Snowling & Melby‐Lervåg, 2016). Similarly, children born into families with a first‐degree relative with language disorder have been found to have significantly poorer language skills at the age of 3 years, compared to children not at family risk, as well as some weaknesses in emergent literacy skills at ages 5 and 7 (Flax et al., 2003). Taken together, this literature provides strong evidence that family history is an important marker of risk of language and literacy difficulties. Evidence from genetically sensitive designs such as twin studies suggests a significant role for genetic factors in this familial transmission, particularly for dyslexia (Olson, Keenan, Byrne, & Samuelsson, 2014), and for language disorder that is accompanied by speech difficulties (Bishop & Hayiou‐Thomas, 2008).

There is substantial evidence that speech and language difficulties in the preschool years are likely to translate into long‐term problems. Several longitudinal studies have reported that young children with identified language disorders are likely to have poorer outcomes in both oral language and literacy than their peers (Beitchman et al., 1994; Catts, Fey, Tomblin, & Zhang, 2002; Stothard et al., 1998; Snowling, Nash, Gooch, Hayiou‐Thomas, & Hulme, 2019). In line with this literature, previous work from the longitudinal Twins Early Development Study (TEDS), the sample that the current study is based on, has found that poor language skills at age 4.5 are moderately predictive of reading at the ages of 7, 9 and 10 (Hayiou‐Thomas, Harlaar, Dale, & Plomin, 2010), and of poor oral language skills at the age of 12 (Hayiou‐Thomas, Dale, & Plomin, 2014).

Speech difficulties (speech sound disorder; SSD) have also been linked to later literacy problems (Bird, Bishop, & Freeman, 1995). This association seems to be strongest when the difficulties are persistent (Bishop & Adams, 1990), when speech errors indicate ‘disordered’ rather than delayed phonology (Leitao & Fletcher, 2004) and particularly when SSD co‐occurs with broader language disorder (Hayiou‐Thomas, Carroll, Leavett, Hulme, & Snowling, 2017; Peterson, Pennington, & Shriberg, 2009), a finding which underlines the importance of considering multiple deficits in the same sample.

The most strongly empirically supported risk markers in early childhood are in the domain of emergent literacy. There is robust, well‐replicated evidence across many different samples and languages that skills such as phonological awareness, letter knowledge and rapid automatized naming are important predictors of subsequent literacy development (Caravolas et al., 2012), and that deficits in these areas are important risk markers for poor literacy outcomes (Kamhi & Catts, 2012).

Finally, and somewhat controversially, is the role of nonverbal factors in language and reading disorders: should nonverbal ability be part of the definition of reading disorders and DLD (Bishop, Snowling, Thompson, Greenhalgh, & CATALISE Consortium, 2016; Siegel, 2003)? We have chosen not to prejudge this question by excluding children with low nonverbal skills; instead, we include this as a risk factor for later difficulties, as suggested by a modest but consistent literature (van Bergen et al., 2014; Chow, Ho, Wong, Waye, & Bishop, 2013; Fuchs et al., 2012). Similarly, our classification of low language and literacy skills in early adolescence is independent of nonverbal level.

Each of the early risk factors discussed above is known to be individually predictive of language and literacy outcomes, but it is not yet clear what their collective contribution is. In part this is because few studies have considered more than one or two of them within the same sample, especially in relation to multiple outcomes, as we do here. It is likely that many of these risk factors reflect overlapping underlying genetic and neurocognitive mechanisms (Pennington & Bishop, 2009). If they are essentially indexing a single underlying dimension of liability, then there should little additional predictive value when including multiple predictors. If they index overlapping but not identical dimensions of risk, then we should see a pattern of cumulative risk.

### 1.2 Relationships among language and literacy outcomes

In the studies reviewed above, early difficulties in spoken language often manifest in later problems in written language, even when the oral language difficulties themselves appear to have resolved (Stothard et al., 1998). Family aggregation and family risk studies strongly suggest that what is shared in the family is not specifically dyslexia or DLD, but rather weak language skills more broadly defined: a child with DLD is as likely to have parents or siblings who have dyslexia, as s/he is to have family members with oral language difficulties (Flax et al., 2003). Similarly, a child with dyslexia is likely to have family members who display weaknesses in spoken language (Snowling & Melby‐Lervåg, 2016). Although they often co‐occur, dyslexia and DLD are not the same disorder: children with dyslexia often have good language skills, and children with DLD may have intact reading (particularly decoding) skills (Bishop, McDonald, Bird, & Hayiou‐Thomas, 2009; Catts, Adlof, & Ellis Weismer, 2006; Snowling et al., 2019). Both the heterogeneity within groups and the overlap between groups sit comfortably within a multiple deficit framework: if there are overlapping liabilities, it may be that some constellations of risk factors are likely to lead to poor language outcomes, others to poor reading fluency and yet others to poor reading comprehension.

### 1.3 The present study

Few studies in this area have included all or most of the identified risk factors, addressed multiple outcomes and covered a substantial developmental range. One aim of the present study is to address these limitations. Another aim is to explicitly address the breadth versus depth question by using early measures in both a categorical (deficit as a unitary concept) or continuous (severity of impairment, in a regression design) fashion.

Research Questions:

1. Does the likelihood of a poor language or literacy outcome increase with the number of risk factors assessed at 4 years?
2. Does including the severity of the early difficulties lead to a better prediction of outcome than a simple count of the number of categorical risk factors?

## 2 METHODS

### 2.1 Participants

The sampling frame for the present study is the Twins Early Development Study (TEDS), a longitudinal study of twins born in England and Wales in 1994, 1995 and 1996. After checking for infant mortality, all families identified by the UK Office for National Statistics (ONS) as having twins born in these years were invited to participate in TEDS when the twins were about 18 months old. The twins have been assessed on measures of language, cognitive and behavioural development from the age of 2 into adulthood, using a variety of methods, including parent questionnaires, telephone testing and web‐based assessment. The TEDS sample has continued to be reasonably representative of the UK population with respect to ethnicity, maternal education and employment and paternal employment (Haworth, Davis, & Plomin, 2013). Informed written consent was obtained from all families participating in TEDS (Institute of Psychiatry ethical approval).

Twin pairs were excluded where either member of the pair had any major medical or perinatal problems, documented hearing loss or organic brain damage. English was the only language spoken at home in all selected families. The sample for the current study was derived from children who had participated in the 4½ (*N* = 1,672) and 12‐year (*N* = 15,038) data collection waves, and was limited to children with complete data on all six risk factors, and data on either language or literacy measures at 12. Only one member of each twin pair was included in analysis to ensure independence of data, yielding a final sample of 210 (103F: 107M). This subsample was slightly upwardly biased in terms of socioeconomic status, as indicated by maternal education: 44.4% of the children had mothers with A‐level qualifications or above (national examinations taken at 18 years of age in the UK). This is similar to the rest of the TEDS sample in adolescence (40.6%) but greater than the national average for children of that generation (35%). Children's mean age at the time of 4‐year data collection was 4.42 years (*SD* = 0.19) and was 12.03 years (*SD* = 0.37) for the 12‐year measures. Further characterization of the sample is provided in Table S1.

### 2.2 Measures

#### 2.2.1 Family history

When children were 9, parents provided information about family history. If any first‐degree relative was reported as having either early language or reading difficulty, we classified this as a positive family history.

Measures of children's language, nonverbal, pre‐literacy and literacy skills were assessed at the ages of 4 and 12, as described below. All composites were created by averaging *z*‐scores that were first corrected for age. Psychometric reliability is reported for all composites included in analyses, and for individual measures where available; we report both Cronbach's alpha as an index of internal reliability and the MZ twin–co‐twin correlation, which provides a lower bound estimate of test–retest reliability.

### 2.3 Child measures at 4

#### 2.3.1 Language composite

An extensive in‐home test battery administered to a subset of TEDS twins at the age of 4 included measures of both receptive and expressive language, assessing lexical, morphological, syntactic and narrative skills (Bus Story Information, Renfrew 1997a; Action Pictures Test Grammar, Renfrew 1997b; Verbal Comprehension, British Ability Scales, Elliot, Smith, & McCulloch, 1996; and Word Knowledge, Verbal Fluency, and Verbal Memory from the McCarthy Scales of Children's Abilities, McCarthy, 1972). Previous analysis has shown that variance across these measures is captured by a single factor, both phenotypically and aetiologically (Hayiou‐Thomas et al., 2006). Based on that finding, we averaged z‐scores for the individual tests to create a composite. This measure shows good internal consistency and reliability, with a Cronbach's alpha calculated from individual test scores of *α* = 0.85, as well as an MZ correlation of *r* = 0.76.

#### 2.3.2 Speech composite

This was computed by averaging two measures in the battery requiring accurate speech production, the Goldman–Fristoe articulation (Goldman & Fristoe, 1986; *α* = 0.96) and nonword repetition (Gathercole, Willis, Baddeley, & Emslie, 1994; *α* = 0.88). These measures had previously been found to be highly intercorrelated, and to load onto a separate factor than the language composite described above (Hayiou‐Thomas et al., 2006). The Cronbach's alpha for this composite is *α* = 0.80, and the lower bound estimate of test–retest reliability based on MZ correlations is *r* = 0.66.

#### 2.3.3 Nonverbal composite

Block Building, Puzzle Solving, Tapping Sequence and Draw‐a‐Design from the McCarthy Scales of Children's Ability (McCarthy, 1972) loaded onto a nonverbal factor (Viding et al., 2003). Cronbach's alpha for this factor is *α* = 0.70, and the MZ correlation is *r* = 0.64.

#### 2.3.4 Phonological awareness

This was indexed by a bespoke 8‐item measure of rhyme judgement, based on a receptive task developed by Bird et al., 1995. Children were introduced to puppets, and asked to point to the picture, out of a choice of four, that sounded like that puppet's name; for example, ‘Which of these things would Dan like?’, ‘Spoon?’, ‘Ring?’, ‘Pan?’, ‘Key?’ (*α* = 0.72).

#### 2.3.5 Letter knowledge

Parents reported on their children's knowledge of letter sounds and names at the age of 4: ‘If shown a letter, can your child name it?’ and ‘If shown a letter, does your child know what sound it makes?’ These two items were combined (Spearman's rho = 0.57).

### 2.4 Child outcome measures at 12

With the exception of the TOWRE, all 12‐year measures were administered online; further information on the testing procedure and validation of this battery is provided in Haworth et al. (2007).

#### 2.4.1 Language composite

Receptive language was assessed at 12 using a battery of audio‐streamed, web‐administered measures indexing vocabulary (WISC‐III‐PI, Kaplan, Fein, Kramer, Delis, & Morris, 1999; *α* = 0.88), syntax (Listening Grammar, Test of Adolescent & Adult Language‐3, Hammill, Brown, Larsen, & Wiederholt, 1994; *α* = 0.94), non‐literal semantics and understanding of inferences (Test of Language Competence‐Level 2, Wiig, Secord, & Sabers, 1989; *α* = 0.66 and *α* = 0.58 respectively). Previous analysis showed substantial phenotypic and genetic overlap among these four measures (Dale, Harlaar, Hayiou‐Thomas, & Plomin, 2010). Cronbach's alpha for this composite *α* = 0.65; the MZ correlation is *r* = 0.66.

#### 2.4.2 Reading fluency composite

Children completed an online adaptation of the Woodcock–Johnson III Reading Fluency test (W‐J III; Woodcock, 2001; *α* = 0.96). In addition, the Test of Word Reading Efficiency, Form B (Torgesen, Wagner, & Rashotte, 1999; alternative forms *r* = 0.73) was included in a test booklet sent to families by mail (one test booklet for each twin), and administered to each twin separately by telephone. Previous work with the TEDS sample at age 7 established strong concurrent validity for telephone administration of the TOWRE (Dale, Harlaar, & Plomin, 2005). The W‐J III and the word and nonword subtests of the TOWRE were combined; Cronbach's alpha for this composite is *α* = 0.82; the MZ correlation is *r* = 0.77.

#### 2.4.3 Reading comprehension composite

Sentence‐level reading comprehension was assessed using a web‐based version of the Reading Comprehension subtest of the Peabody Individual Achievement Test (PIAT; Markwardt, 1997; *α* = 0.94), in which children read a sentence and chose the matching picture from a set of four. In addition, children completed a web version of the GOAL Formative Assessment in Literacy for Key Stage III (GOAL plc, 2002; *α* = 0.91), which includes a wide range of literal and inferential comprehension questions based on a set of stimulus sentences and short paragraphs. These measures were combined; Cronbach's alpha for this composite is *α* = 0.67; the MZ correlation is *r* = 0.64.

### 2.5 Defining risk factors and outcomes

A key aim of the current study is to contrast the predictive value of using categorical versus continuous measures of risk. With the exception of family risk, which was coded as present or absent, the risk factors we examine at 4 are based on continuous measures, so cut‐off criteria had to be determined for the categorical analysis. Based on common practice in studies of early vocabulary (see, e.g. multiple studies included in Rescorla & Dale, 2013), we chose a criterion of – 1.25 *SD* for the language, speech and nonverbal composites and the phonological awareness measure, and falling below the 10th centile on the letter knowledge measure, given its high skewness.

With respect to the outcomes of interest, it is increasingly acknowledged that the great majority of language and literacy impairments are best thought of as lying on a continuum of language ability, rather than reflecting a qualitatively different category. For this reason, cut‐offs are inherently somewhat arbitrary. Nevertheless, they are essential clinically for decisions concerning service delivery, and essential in research to focus attention on the interplay of factors at the low end of the continuum, which may differ from that in the centre or high end. We have chosen a broad criterion for defining poor language, reading fluency and reading comprehension outcomes, based on scoring at least −1 *SD* below the mean on the relevant composite. This cut‐off was chosen both because it has been widely used in previous research (e.g. language: Armstrong et al., 2018; Rice, Taylor, & Zubrick, 2008; Snowling, Duff, Nash, & Hulme, 2016; dyslexia: Catts, Adlof, Hogan, & Weismer, 2005; poor reading comprehension: Nation, Clarke, Marshall, & Durand, 2004), and also to maximize the sample of children with weak language or literacy skills at 12.

### 2.6 Analysis

To address the first research question, concerning the relation of number of risk factors with poor outcomes, the primary analysis method is the computation of Odds Ratios. Odds Ratios are the accepted benchmark in medical research, because they provide a measure of association between a risk factor and the outcome of interest by comparing the odds of a particular outcome occurring when the risk is present, to the odds of that outcome occurring in the absence of risk. In addition to Odds Ratios, we also computed Spearman's correlations between the number of risk factors and each of the three outcomes. The second research question is addressed by comparison of the accuracy of predictions based on dichotomizing a simple count of risk factors with logistic regressions based on continuous measures of risk factors. In each case, a criterion was set for prediction of poor outcome, which approximately matched the actual proportion in the sample. Logistic regressions were calculated with continuous measures of risk factors, and the residual ‘estimated probability of positive outcome’ was saved and dichotomized. Four conventional measures of diagnostic validity were then calculated: sensitivity, specificity, positive predictive value (PPV) and negative predictive value (NPV). In addition, ROC analysis was carried out based on these models, and the Area Under the Curve statistic was used to indicate overall predictive validity independent of a specific criterion.

## 3 RESULTS

### 3.1 Outcomes

**FIGURE 1:**
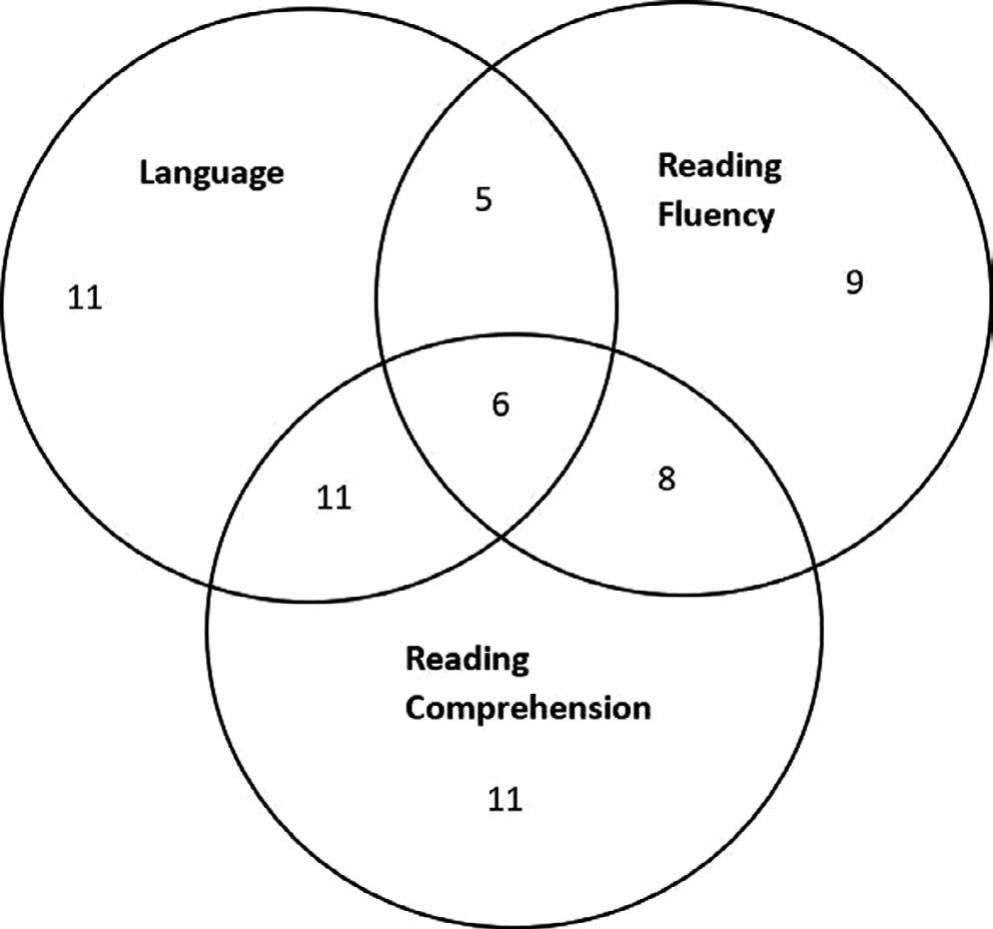
Overlap between poor outcomes in oral language, reading fluency and reading comprehension (*N*), for the subset of children for whom we had data on all three outcomes (*N* = 163), and who met criteria for at least one poor outcome at 12 (*N* = 61). Note: a small number of additional children, who were included in the main analyses but not this figure, had a poor outcome in one domain, but were missing data for the other outcomes (language *N* = 1, reading fluency *N* = 6, reading comprehension *N* = 2)

Of the 210 children in our sample, 34 (13F: 21M) met criteria for a poor outcome in oral language at 12, 34 (13F: 21M) in reading fluency and 38 in reading comprehension (14F: 24M). Several children met criteria for more than one outcome: this overlap is illustrated in the Venn diagram in Figure 1, for the subset of children for whom we had data on all three outcomes (*N* = 163), and who met criteria for at least one poor outcome at 12 (*N* = 61).

### 3.2 Risk factors

Approximately 40–50 children (20%–25% of the sample) met criteria for each of the individual risk factors, with a much smaller proportion (2%–7%) meeting criteria for *only* that risk factor (Table 1). We used the cumulative risk index (min:max 0–6) to examine the distribution of risk factors in our sample. Eighty‐nine (42%) of the 210 children in the sample did not have any risk factors present at 4, and as expected, in general, the greater the number of risk factors, the fewer the cases: 45 children (21%) had one risk factor, 27 (13%) had two, 34 (16%) had three, 8 (4%) had four and 7 (3%) had five. No children had all six risk factors. The risk factors were modestly associated with one another, as indicated by the phi correlations presented in Table 2.

**TABLE 1:** Occurrence of individual risk factors in the overall sample (*N* = 210)

Risk factor at 4	*N* (%) of children with this risk factor at 4 (categorical)	*N* (%) of children with ONLY this risk factor at 4 (categorical)	Mean level (*M*; *SD*) on this measure at 4, for the whole sample (continuous)
Family risk	56 (26.67%)	15 (7.14%)	56 (26.67%)
Language	47 (22.38%)	5 (2.38%)	−0.34 (1.16)
Speech	42 (20.00%)	4 (1.90%)	−0.26 (1.15)
Phonological awareness	40 (19.05%)	8 (3.81%)	−0.32 (1.03)
Letter knowledge	46 (21.90%)	7 (3.33%)	1.70 (1.17)
Nonverbal ability	37 (17.62)%	6 (2.86%)	−0.23 (1.16)

**TABLE 2:** Phi correlations among risk factors

	Language	Speech	Phonological Awareness	Letter Knowledge	Nonverbal
Family risk	0.167*	0.237**	0.091	0.175*	0.004
Language		0.246**	0.089	0.351**	0.351**
Speech			0.273**	0.311**	0.269**
Phonological awareness				0.124	0.126
Letter knowledge					0.208**

* *p* < .05;

** *p* < .01.

#### 3.2.1 Number of risk factors and the likelihood of a poor outcome (research question 1)

Spearman's correlations indicated that there was a modest but significant (*p* < .001) linear relationship between the number of risk factors present at age 4, and outcomes at 12: Language *r* = 0.36, Reading fluency *r* = 0.32; Reading comprehension *r* = 0.40. These relationships remained significant when including only the subset of cases with at least one risk factor, that is, within the group having some degree of risk: Language *r* = 0.19 (*p* = .050), Reading fluency *r* = 0.26 (*p* = .004); Reading Comprehension *r* = 0.32 (*p* < .001).

**FIGURE 2:**
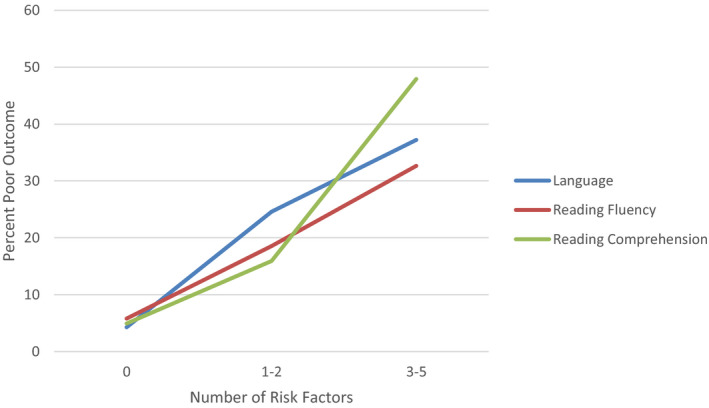
Proportion of children with poor outcomes in three domains as a function of number of risk factors

Odds ratios were used to estimate the odds of a poor outcome in each domain in the presence of either few (1–2) or many (3–6) risk factors, relative to the odds of a poor outcome when no risk factors were present. Table 3 shows the proportion of children in each of these ‘no’, ‘low’ or ‘high‐risk’ groups who had a poor outcome at the age of 12; the overall pattern is illustrated in Figure 2. Only a small proportion of children who had no risks at all at 4 went on to meet our criteria for a poor outcome in oral language (4.28%), reading fluency (5.81%) or reading comprehension (4.94%) at age 12. A larger proportion of the group with 1–2 risk factors had poor outcomes (roughly 15%–25%), and a larger proportion still in the group with 3–6 risk factors (roughly 35%–45%).

**TABLE 3:** Number (%) of children with poor outcomes in each domain, according to the number of risk factors; and associated Odds Ratios

	Good outcome (*N*)	Poor outcome (*N*)	Total (*N*)	% poor outcome	Odds ratios (95% CI) Chi‐sq (*df*, *p*‐value)	
Oral language						
0 risk	67	3	70	4.28%		
1–2 risks	46	15	61	24.59%	7.28 (1.99–26.59)	11.34 (*df* = 1, *p* = .001)
3–6 risks	27	16	43	37.21%	13.24 (3.56–49.13)	20.64 (*df* = 1, *p* < .001)
*TOTAL N*	140	34	174	19.54%		
Reading fluency						
0 risks	81	5	86	5.81%		
1–2 risks	57	13	70	18.57%	3.70 (1.25–10.94)	6.15 (*df* = 1, *p* = .013)
3–6 risks	33	16	49	32.65%	7.86 (2.66–23.19)	17.12 (*df* = 1, *p* < .001)
*TOTAL N*	171	34	205	16.58%		
Reading comprehension						
0 risk	77	4	81	4.94%		
1–2 risk	58	11	69	15.94%	3.65 (1.11–12.05)	5.01 (*df* = 1, *p* = .025)
3–6 risks	25	23	48	47.92%	17.71 (5.59–56.14)	33.64 (*df* = 1, *p* < .001)
*TOTAL N*	160	38	198	19.19%		

The odds ratios confirm that the presence of at least some risk factors is significantly associated with a poor language or reading outcome, with ORs ranging from 3.65 to 17.71 (Table 3). It is also notable that for all three outcomes, the odds ratios are substantially larger when many risk factors are present, as compared to one or two, although the overlapping confidence intervals between 1–2 risks and 3–6 risks show that these odds ratios are not statistically different from each other with this sample size. However, the existence of the overall association between number of risk factors and poor outcomes is supported by the positive and significant correlations between them reported above.

#### 3.2.2 Does depth add to breadth with respect to predictive accuracy? (research question 2)

Having established an association between number of early risks and later outcomes, we examined the utility of the early measures for predicting risk at the level of the individual child. Specifically, we compared two types of models in which different levels of information about the predictor variables were included. In the first, only the presence or absence of each risk factor was considered. The second type of model made use of continuous rather than categorical measures of the risk factors, as well as allowing differential weight of predictors in logistic regressions. Table 4 summarizes and compares the main results of the analyses, particularly diagnostic validity for the models in terms of sensitivity, specificity and positive and negative predictive value.

**TABLE 4:** Comparison of the accuracy, including confidence intervals, of two predictive models of poor outcomes in oral language, reading fluency and reading comprehension

Prediction model	Outcome at 12	Criterion^#^	Sensitivity	Specificity	Positive predictive value	Negative predictive value	AUC (95% CI)
Number of risk factors	Oral language	≥3	0.47 (0.30–0.65)	0.81 (0.73–0.87)	0.37 (0.27–0.49)	0.86 (0.82–0.90)	0.75*a (0.66–0.84)
	Reading fluency	≥3	0.47 (0.30–0.65)	0.81 (0.74–0.863)	0.33 (0.23–0.44)	0.88 (0.85–0.91)	0.74*b (0.65–0.83)
	Reading comprehension	≥3	0.61 (0.43–0.76)	0.84 (0.78–0.90)	0.48 (0.37–59)	0.90 (0.86–0.93)	0.78*c (0.70–0.86)
Continuous measures of risk factors	Oral language	0.370	0.50 (0.32–0.68)	0.89 (0.83–0.94)	0.53 (0.39–0.67)	0.88 (0.84–0.91)	0.84*a (0.66–0.84)
	Reading fluency	0.339	0.53 (0.35–0.70)	0.91 (0.859–0.95)	0.55 (0.40–0.68)	0.91 (0.87–0.93)	0.77*b (0.68–0.87)
	Reading comprehension	0.340	0.55 (0.38–0.71)	0.89 (0.84–0.94)	0.55 (0.42–0.68)	0.89 (0.85–0.92)	0.83*c (0.75–0.90)

^#^ For the second model, the criterion is the critical value of the logistic residual ‘estimated probability of positive outcome; values ≥ the criterion are predictions of poor outcome.

* All AUC statistics had asymptotic significance < 0.001. The AUC values for each pair of models (categorical vs. continuous risk) for the three outcomes were formally compared using a critical ratio *z* value. These indicated no significant differences: ^a^ *z* = 0.32 *p* = .626; ^b^z = 0.10, *p* = .540; ^c^z = 0.18, *p* = .571.

**FIGURE 3:**
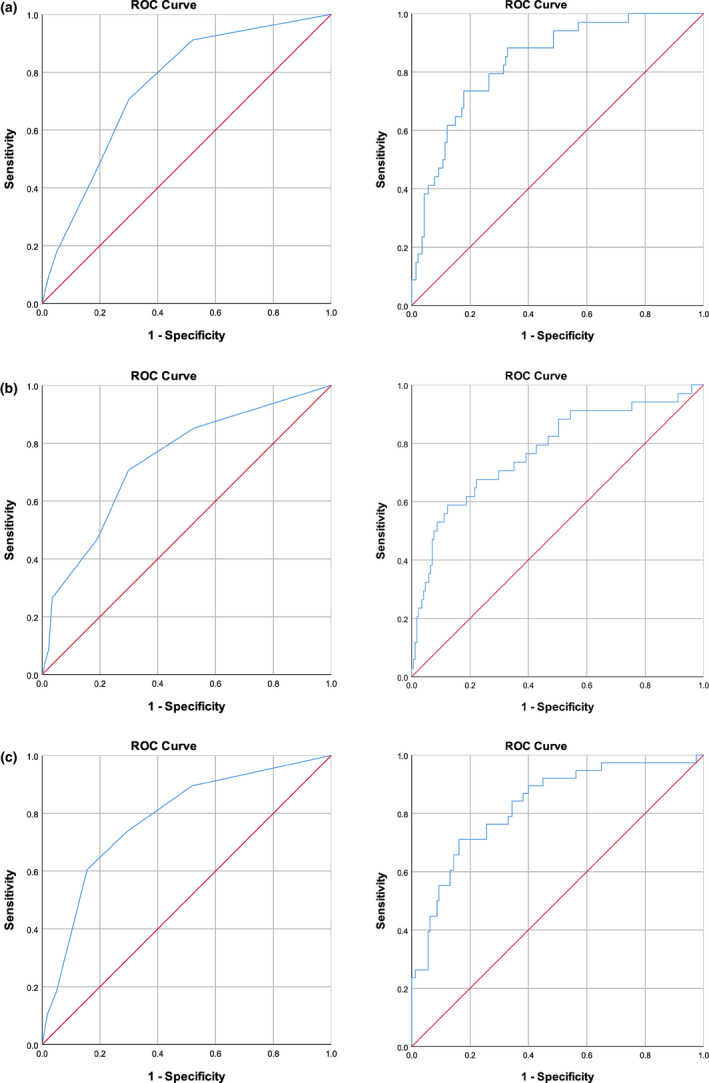
Area Under the Curve for prediction from the Cumulative Risk Index (categorical predictors; left panels) and the probability estimated from the logistic regression analysis (continuous predictors; right panels). (a) Poor language outcome. (b) Poor reading fluency outcome. (c) Poor reading comprehension outcome

We also report the results of a receiver operating characteristic (ROC) curve analysis, which provides a more comprehensive assessment of the validity of prediction because it is not tied to any specific cut‐off of risk. Instead, it plots sensitivity against specificity for each possible predictor cut‐off. The area under the curve (AUC) statistic is a measure of the extent to which the ROC curve diverges from the diagonal, which indicates chance performance. ROC curves are presented in Figure 3, and the associated AUC statistics are included in Table 4.

Finally, we report the results for individual predictors in Table 5. The Odds Ratios show the level of risk associated with each predictor (regardless of the presence of other risk factors), when this is defined categorically. The B weights are derived from the logistic regression model (Model 2, described below), in which the predictors are continuous measures.

**TABLE 5:** Effects of individual risk factors on poor outcomes in three domains, as indexed by odds ratios for categorically defined risks (top row for each outcome), and logistic regression weights (B and S.E.) derived from the continuous risk factors analysis (bottom row)

Outcome at 12	Analysis type and statistic	Family risk	Language	Speech	Phonological awareness	Letter knowledge	Non verbal
Oral language	Odds ratio (95% CI)	2.56 (1.17–5.59)	6.44 (2.86–14.50)	1.92 (0.82–4.51)	1.23 (0.50–3.01)	2.99 (1.32–6.79)	5.03 (2.19–11.55)
	B (S.E.)	−0.818 (0.480)	−1.118** (0.342)	−0.045 (0.259)	0.448 (0.278)	0.239 (0.252)	−0.633* (0.280)
Reading fluency	Odds ratio (95% CI)	4.75 (2.20–10.26)	3.43 (1.57–7.47)	3.73 (1.68–8.28)	1.69 (0.72–3.99)	2.59 (1.18–5.70)	2.67 (1.16–6.12)
	B (S.E.)	−1.288** (0.424)	−0.470 (0.282)	−0.422 (0.239)	−0.015 (0.241)	0.073 (0.224)	−0.103 (0.258)
Reading comprehension	Odds ratio (95% CI)	2.34 (1.11–4.89)	6.37 (2.96–13.68)	4.59 (2.12–9.94)	1.61 (0.71–3.69)	1.78 (0.81–3.89)	10.00 (4.40–22.70)
	B (S.E.)	−0.760 (0.453)	−0.695* (0.283)	−0.129 (0.242)	−0.162 (0.254)	0.427 (0.245)	−0.903** (0.275)

Note

Within each type of analysis, comparison across risk factors (columns) indicates the relative prediction weight of each factor. Because of differing assumptions in the two types of analysis, direct comparisons across rows (odds ratios vs. B weights) are not appropriate.

* *p* < .05.

** *p* < .01.

### 3.3 Model 1: Cumulative risk

The diagnostic validity measures for this model, which essentially weights all risk factors at the same level, were derived by dichotomizing the number of risk factors (0–2 and 3–6). This cut‐point was selected because it yielded the smallest high‐risk sample (23.3%) that was at least as large as the proportion actually observed at 12 years for a poor outcome (19.5% for language, 16.6% for reading fluency and 19.2% for reading comprehension). For all three outcomes, this model yielded moderate overall prediction, although it was notably poor with respect to sensitivity and positive predictive value at the selected cut‐point (Table 4). The AUC values ranged between 0.74 and 0.78, which can be considered ‘fair’ classification values (Caspi, Houts, Belsky, & Harrington, 2016).

### 3.4 Model 2: Continuous measures with differential weighting

A set of logistic regressions was run with the underlying continuous measures for all predictors except family history (which is inherently categorical). Thus, unlike Model 1 in which the same risk index (number of risk factors) was used for all three outcomes, in Model 2, a separate risk index was calculated for each outcome based on the logistic regression analysis for that outcome. As an indication of potential for differential prediction, correlations were computed between number of risk factors and each of the three new indices. They were 0.66, 0.81 and 0.71 for language, reading fluency and reading comprehension respectively. The correlation for reading fluency was significantly higher than that for the other two outcomes, using the *r*‐to‐*z* transformation evaluation procedure. Although these correlations were substantial, they were still low enough to allow for differential prediction of outcomes. Overall, the values of the classification indices for Model 2 were similar to those of Model 1, although there was a slight increase in positive predictive value, and in the AUC statistics (AUC 0.78–0.84; values of 0.80 and above can be considered ‘good’, Caspi et al., 2016). Taken together, these results show only modest improvement in prediction over cumulative risk even when full (continuous) information about the predictors is utilized and separate prediction formulas are used.

The ROC analysis allows a formal evaluation of the change in prediction from Model 1 to Model 2. The overlapping 95% confidence intervals for the AUC statistics in Table 4 indicated that for none of the three outcomes was the area under the curve significantly different between the analysis based on the cumulative risk index and that based on the estimated probability from the logistic regression. This is further confirmed by the non‐significant critical ratio z‐values for each pair of models (Hanley & McNeil, 1983).

In order to test the robustness of our findings that categorical versus continuous models yield similar predictive values, we carried out a supplementary analysis using a different statistical approach (reported in Supplementary Online Materials 2). Classification and regression tree (CART) models produce an easily interpretable visual output which shows the predicted outcome for individual children based on their score on each predictor variable (Figures [Link], [Link], [Link]). They also produce classification tables which can be used to derive diagnostic indices (sensitivity/specificity/PPV/NPV; Table S2). Applied to our data, CART models yielded overall comparable prediction of outcome for categorical versus continuous predictors: of the 12 pairs of diagnostic validity indices, only one had non‐overlapping confidence intervals (the sensitivity index for Reading Fluency).

Examining the individual predictors (Table 5), it can be seen that family history is a significant predictor of a poor reading fluency outcome at 12; that both early language and early nonverbal skills are significant predictors of a poor language outcome and that early language and nonverbal ability are again the only significant predictors of a poor reading comprehension outcome. This pattern of results is supported both by the logistic regressions for Model 2, which use continuous predictors, and by the Odds Ratios for individual risk factors defined categorically: the risk factors which were significant in the logistic regressions were also the ones which had the largest Odds Ratios for each outcome.

## 4 DISCUSSION

Our results show that the presence of a greater number of risk factors at the age of 4 increases the likelihood of a poor outcome in language and literacy at 12. Somewhat surprisingly, we find that adding information about the severity of the early predictors, and allowing the weighting of individual predictors to vary, adds little predictive value. At the same time, although the pattern of increased likelihood of a poor outcome associated with ‘cumulative risk’ is clear, we also show that even with rich information in early childhood, it is still not easy to predict which children will have a poor outcome at the age of 12.

### 4.1 Breadth versus depth in three outcome domains

Both of these aspects of our findings—the predominant role of breadth, and moderate individual prediction—cohere with, and extend, the existing literature. Previous studies have shown that the presence of additional early deficits is associated with poorer outcomes: children with language difficulties combined with poor nonverbal ability are more likely to have poor language and literacy outcomes in later childhood and adolescence (Bishop & Adams, 1990; Stothard et al., 1998). Similarly, speech deficits accompanied by language difficulties in preschool are more likely to be associated with poor literacy outcomes than isolated speech disorder (Peterson et al., 2009), and even more likely if there is also a family risk of dyslexia (Carroll, Mundy, & Cunningham, 2014; Hayiou‐Thomas et al., 2017). Children at family risk of dyslexia are in turn more likely to meet criteria for dyslexia if they also have weak language skills in early childhood (Snowling & Melby‐Lervag, 2016). Although in the current study, we have not delineated the specific combinations of risk factors which are associated with specific outcomes, we have demonstrated that this general pattern of cumulative risk seems to hold across a broad range of language‐relevant predictors and outcomes.

By itself, a correlation between number of risk factors and poor outcome is consistent with a depth interpretation; as the early predictors are often intercorrelated, they collectively might be indexing a single underlying dimension of risk, that is, breadth might be a proxy for depth. The failure to significantly improve prediction when (in Model 2) severity measures are included in the prediction equation provides support for the conclusion that the predictor variables here do primarily constitute measures of breadth of risk.

### 4.2 Prediction of poor outcomes from early measures

Turning to the prediction of individual risk, we found moderate levels of overall prediction over the long range from ages 4 to 12. The AUC values our models yielded were either just below or just above 0.80, which is at the lower end of what is considered an adequate value for clinical decision‐making about intervention (Caspi et al., 2016). This level of prediction is in line with other findings in the literature. For example, in predicting 7‐year outcomes in receptive language from 4‐year‐old language scores, McKean et al. (2017) reported an AUC value of 0.84, while in a study predicting reading disability at age 8 from pre‐literacy measures at ages 3 and 5, Puolakanaho et al. (2007) reported AUC values from 0.70 to 0.85. Interestingly, potentially more powerful statistical approaches utilizing methods which can take account of complex interactions among specific combinations of predictors do not find that these enhance the levels of prediction for language or reading outcomes (e.g. classification and regression tree [CART] models, Koon, Petscher, & Foorman, 2014; and machine learning based on neural net modelling, Armstrong et al., 2018).

This challenge in accurate long‐range prediction of language outcomes for individual children occurs despite high levels of stability in language and literacy skills in terms of individual variability, at least after the age of 5 (e.g. Bornstein, Hahn, & Putnick, 2016; Norbury et al., 2017), as well as reasonable levels of stability in diagnostic status, particularly when effects of regression to the mean are taken into account (Johnson et al., 1999; Tomblin, Zhang, Buckwalter, & O’Brien, 2003). Nonetheless, it is also the case that for many children, early language difficulties resolve over time, while for other children, language difficulties emerge later in childhood (Hayiou‐Thomas et al., 2014; Snowling et al., 2015). Reading difficulties also appear to be stable over the short term, with dyslexia in Grade 1 predicting dyslexia in Grade 2 with a PPV of 68.3% (Pennington et al., 2012), but the long‐range prediction of poor reading outcomes from pre‐reading skills is still challenging. It is possible that an even broader range of predictors than ours needs to be considered, for example, executive function (Thompson et al., 2015) or sensory and motor deficits (Carroll, Solity, & Shapiro, 2016).

### 4.3 Limitations and future directions

An important limitation to bear in mind when interpreting our findings is that of statistical power and sample size. Our initial sampling frame is from the large‐scale TEDS sample, and we had direct measures of speech and language at the age of 4 on a subset of 1672 children. Attrition over 8 years, our requirement of a broad range of both predictor and outcome measures, and the need to utilize only one twin from each pair to ensure independence of data reduced this sample to 210. While sufficient for addressing our main research questions, this sample size prevented us from examining the data at a more fine‐grained level, for example, comparing the contribution of individual predictors and specific combinations of predictors (there are 63 possible combinations of six predictors). The cumulative pattern of risk that we observed in our data is suggestive of an additive accumulation of risk, but it will also be important to explore the possibility of interactions among specific risk factors. That is, are there specific constellations of risk factors that are more likely to give rise to a specific outcome? And, if so, what are the causal pathways that mediate the relationship between sets of early risks and later outcomes, an issue that our correlational design cannot address?

We also note that in this study, we did not have a measure of Rapid Automatized Naming, which is an established predictor of reading (Caravolas et al., 2012), and our measure of letter knowledge was non‐standard, in that it was based on parental report of the level of letter knowledge, rather than a direct assessment of knowledge of each letter name or sound. In addition, our measure of phonological awareness focused on rhyme, but it is likely that phoneme awareness specifically is a more sensitive predictor than onset–rime analysis, which was assessed in the current study (Hulme et al., 2002).

Finally, we acknowledge that interpretation of results from (non‐experimental) longitudinal studies of language disorders is particularly challenging, given the potential role of intervention services. In the present study, information is available from only a subsample of the families, and it is limited to the provision of service, not the target of services, intensity and duration of service or therapeutic approach utilized. But even if more complete information were available, there is the problem that level of language ability is causally both an antecedent and a consequent of service provision. For this reason, comparison of language between children who have received services and those who have not is inherently ambiguous.

### 4.4 Clinical implications

Despite these limitations, the current study has clinical implications for the identification of children with language and literacy disorders. Firstly, the greater the number of early risk factors, the greater the likelihood of a poor outcome: that is, breadth matters. Secondly, and more surprisingly, the severity of early deficits may not in itself be very informative: depth adds little to prediction beyond breadth. And thirdly, it is important to be aware that difficulties may emerge in later childhood despite the absence of red flags in the early years.

## CONFLICT OF INTEREST

No conflicts declared.

## Supporting information

Figure S1a

Figure S1b

Figure S2a

Figure S2b

Figure S3a

Figure S3b

Table S1

Table S2

## Data Availability

Data used for this submission may be made available on request to the Twins Early Development Study (TEDS), through their data access mechanism (see www.teds.ac.uk/research/collaborators‐and‐data/teds‐data‐access‐policy). They will then consider requests for sharing data for appropriate research purposes.
