# Supplementary figures and images for: Breadth versus depth: Cumulative risk model and continuous measure prediction of poor language and reading outcomes at 12
Source: Dev Sci. 2020 Jun 22;24(1):e12998. doi: 10.1111/desc.12998 (PMC11475567; doi:10.1111/desc.12998)

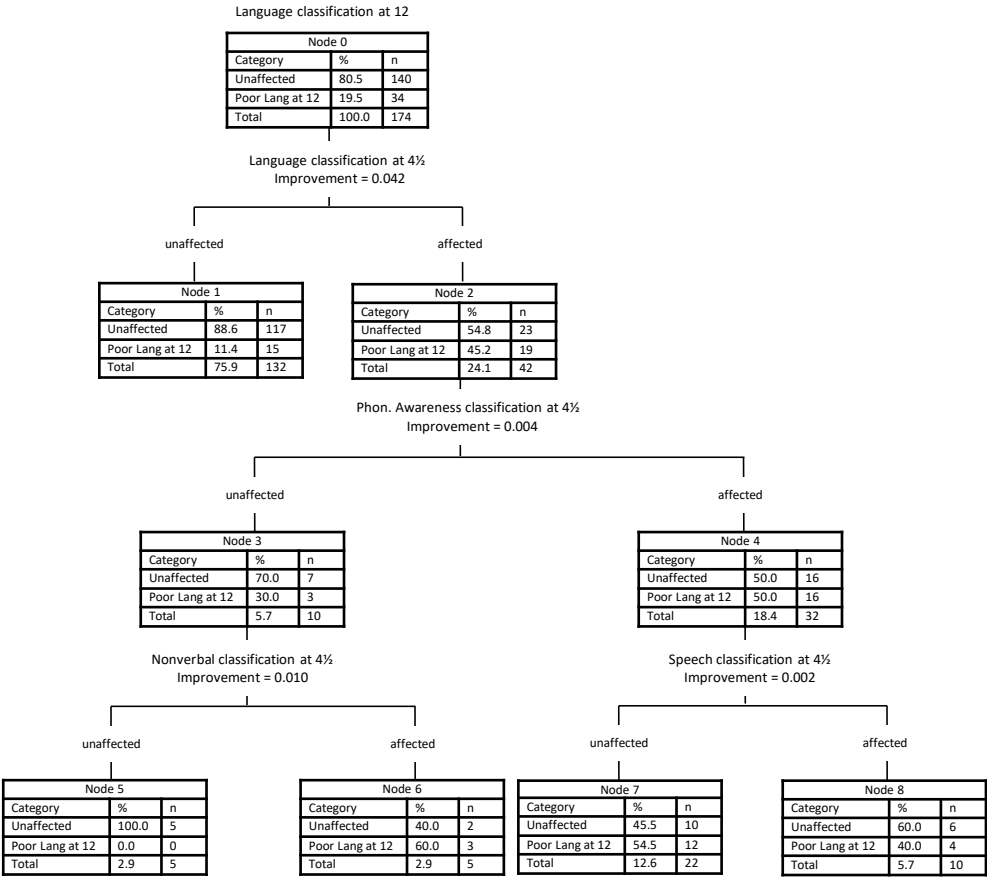

Supplement: Supplementary file 1 — Figure S1a [file DESC-24-e12998-s007.pdf]

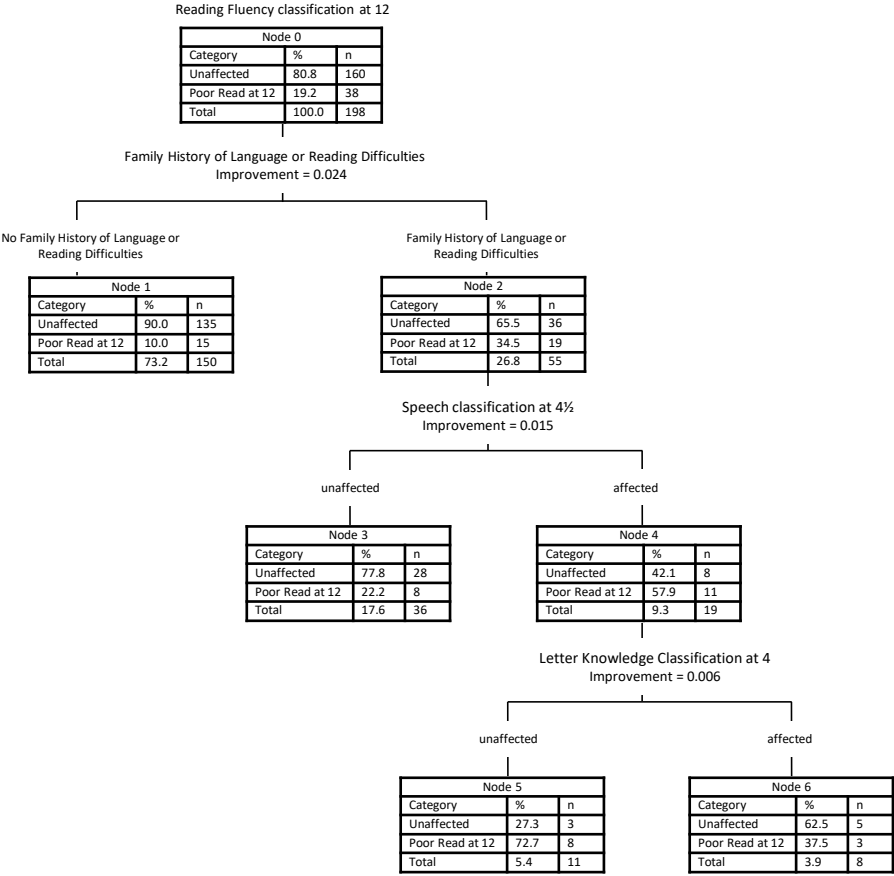

Supplement: Supplementary file 3 — Figure S2a [file DESC-24-e12998-s005.pdf]

Reading Fluency classification at 12

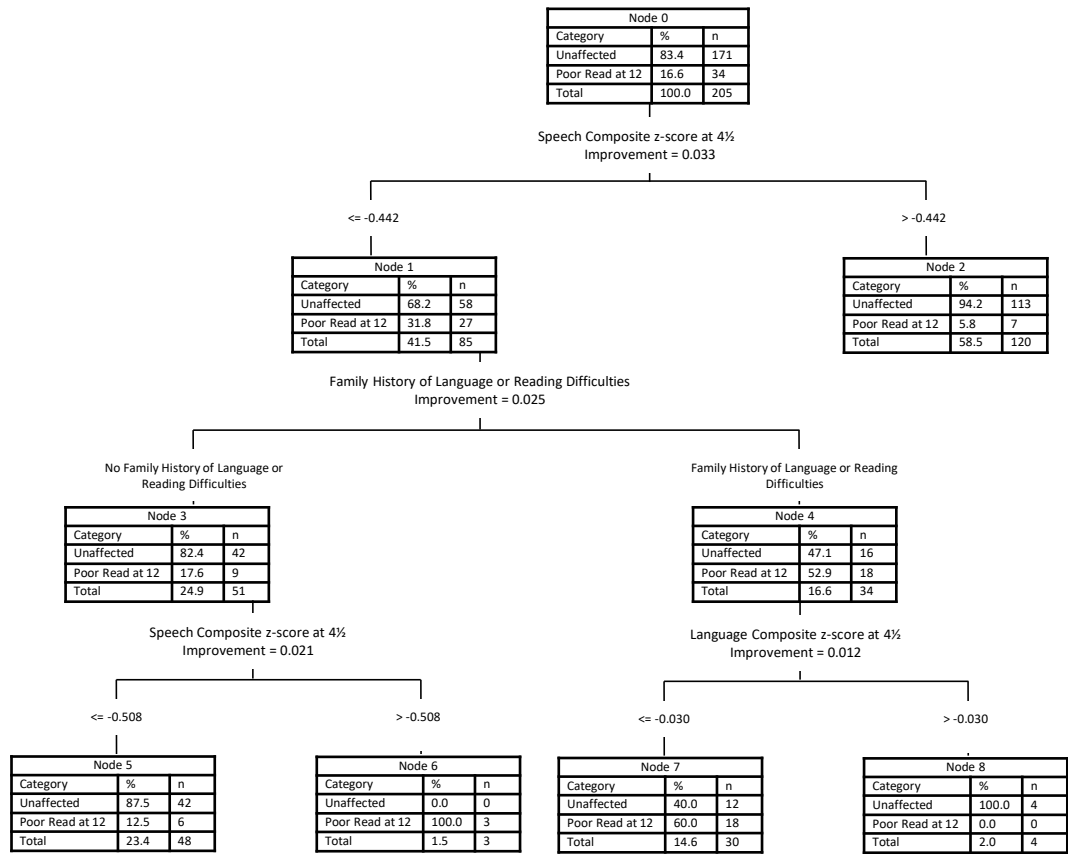

Supplement: Supplementary file 4 — Figure S2b [file DESC-24-e12998-s004.pdf]
