# Supplementary material for: Breadth versus depth: Cumulative risk model and continuous measure prediction of poor language and reading outcomes at 12
Source: Dev Sci. 2020 Jun 22;24(1):e12998. doi: 10.1111/desc.12998 (PMC11475567; doi:10.1111/desc.12998)
Supplement: Supplementary file 2 — Figure S1b [file DESC-24-e12998-s006.pdf]

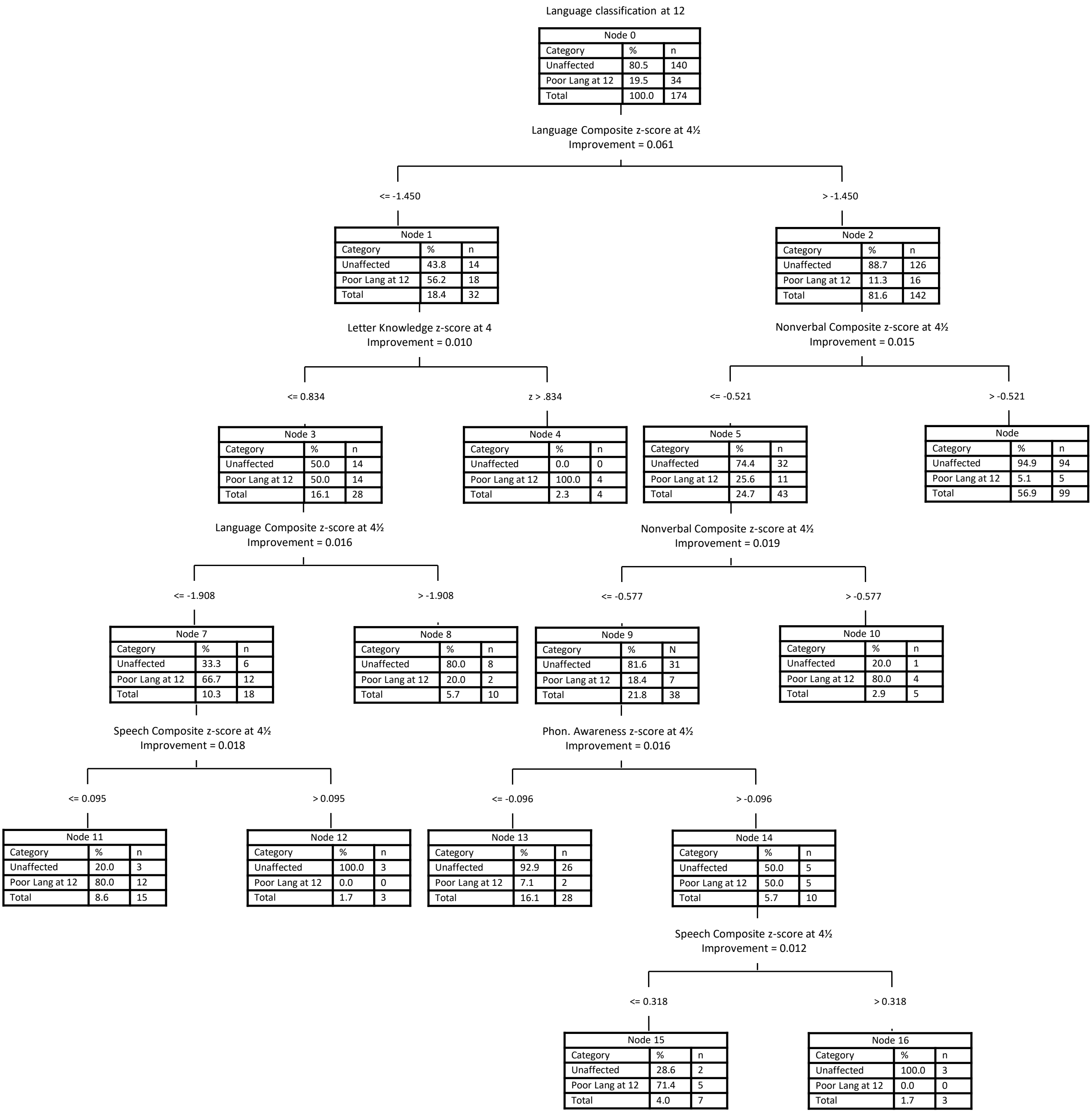
Language classification at 12
Node 0
Category % n
Unaffected 80.5 140
Poor Lang at 12 19.5 34
Total 100.0 174
Language Composite z-score at 4½
Improvement = 0.061
<= -1.450
> -1.450
Node 1
Category % n
Unaffected 43.8 14
Poor Lang at 12 56.2 18
Total 18.4 32
Node 2
Category % n
Unaffected 88.7 126
Poor Lang at 12 11.3 16
Total 81.6 142
Letter Knowledge z-score at 4
Improvement = 0.010
Nonverbal Composite z-score at 4½
Improvement = 0.015
<= 0.834
z > .834
<= -0.521
> -0.521
Node 3
Category % n
Unaffected 50.0 14
Poor Lang at 12 50.0 14
Total 16.1 28
Node 4
Category % n
Unaffected 0.0 0
Poor Lang at 12 100.0 4
Total 2.3 4
Node 5
Category % n
Unaffected 74.4 32
Poor Lang at 12 25.6 11
Total 24.7 43
Node
Category % n
Unaffected 94.9 94
Poor Lang at 12 5.1 5
Total 56.9 99
Language Composite z-score at 4½
Improvement = 0.016
Nonverbal Composite z-score at 4½
Improvement = 0.019
<= -1.908
> -1.908
<= -0.577
> -0.577
Node 7
Category % n
Unaffected 33.3 6
Poor Lang at 12 66.7 12
Total 10.3 18
Node 8
Category % n
Unaffected 80.0 8
Poor Lang at 12 20.0 2
Total 5.7 10
Node 9
Category % N
Unaffected 81.6 31
Poor Lang at 12 18.4 7
Total 21.8 38
Node 10
Category % n
Unaffected 20.0 1
Poor Lang at 12 80.0 4
Total 2.9 5
Speech Composite z-score at 4½
Improvement = 0.018
Phon. Awareness z-score at 4½
Improvement = 0.016
<= 0.095
> 0.095
<= -0.096
> -0.096
Node 11
Category % n
Unaffected 20.0 3
Poor Lang at 12 80.0 12
Total 8.6 15
Node 12
Category % n
Unaffected 100.0 3
Poor Lang at 12 0.0 0
Total 1.7 3
Node 13
Category % n
Unaffected 92.9 26
Poor Lang at 12 7.1 2
Total 16.1 28
Node 14
Category % n
Unaffected 50.0 5
Poor Lang at 12 50.0 5
Total 5.7 10
Speech Composite z-score at 4½
Improvement = 0.012
<= 0.318
> 0.318
Node 15
Category % n
Unaffected 28.6 2
Poor Lang at 12 71.4 5
Total 4.0 7
Node 16
Category % n
Unaffected 100.0 3
Poor Lang at 12 0.0 0
Total 1.7 3
