# Supplementary material for: Breadth versus depth: Cumulative risk model and continuous measure prediction of poor language and reading outcomes at 12
Source: Dev Sci. 2020 Jun 22;24(1):e12998. doi: 10.1111/desc.12998 (PMC11475567; doi:10.1111/desc.12998)
Supplement: Supplementary file 5 — Figure S3a [file DESC-24-e12998-s001.pdf]

Reading Comprehension classification at 12

Node 0		
Category	%	n
Unaffected	80.8	160
Poor RC at 12	19.2	38
Total	100.0	198

Nonverbal Classification at 4½
Improvement = 0.059

affected

Node 1		
Category	%	n
Unaffected	44.4	16
Poor RC at 12	55.6	20
Total	18.2	36

Family History of Language or Reading Difficulties
Improvement = 0.008

No Family History of Language or Reading Difficulties

Node 3		
Category	%	n
Unaffected	53.8	14
Poor RC at 12	46.2	12
Total	13.1	26

Language Classification at 4½
Improvement = 0.015

unaffected

Node 5		
Category	%	n
Unaffected	33.3	5
Poor RC at 12	66.7	10
Total	7.6	15

affected

Node 6		
Category	%	n
Unaffected	81.8	9
Poor RC at 12	18.2	2
Total	5.6	11

Family History of Language or Reading Difficulties

Node 4		
Category	%	n
Unaffected	20.0	2
Poor RC at 12	80.0	8
Total	5.1	10

unaffected

Node 2		
Category	%	n
Unaffected	88.9	144
Poor RC at 12	11.1	18
Total	81.8	162
