# Supplementary material for: Breadth versus depth: Cumulative risk model and continuous measure prediction of poor language and reading outcomes at 12
Source: Dev Sci. 2020 Jun 22;24(1):e12998. doi: 10.1111/desc.12998 (PMC11475567; doi:10.1111/desc.12998)
Supplement: Supplementary file 6 — Figure S3b [file DESC-24-e12998-s008.pdf]

Reading Comprehension classification at 12

Node 0		
Category	%	n
Unaffected	80.8	160
Poor RC at 12	19.2	38
Total	100.0	198

Nonverbal Composite z-score at 4½
Improvement = 0.078

<= -1.062

Node 1		
Category	%	n
Unaffected	44.4	20
Poor RC at 12	55.6	25
Total	22.7	45

Language Composite z-score at 4½
Improvement = 0.027

<= -1.486

Node 3		
Category	%	n
Unaffected	15.8	3
Poor RC at 12	84.2	16
Total	9.6	19

> -1.486

Node 4		
Category	%	n
Unaffected	65.4	17
Poor RC at 12	34.6	9
Total	13.1	26

> -1.062

Node 2		
Category	%	n
Unaffected	91.5	140
Poor RC at 12	8.5	13
Total	77.3	153
