# Supplementary material for: Breadth versus depth: Cumulative risk model and continuous measure prediction of poor language and reading outcomes at 12
Source: Dev Sci. 2020 Jun 22;24(1):e12998. doi: 10.1111/desc.12998 (PMC11475567; doi:10.1111/desc.12998)
Supplement: Supplementary file 7 — Table S1 [file DESC-24-e12998-s002.docx]

|  | **Construct** | **Whole Sample**  **(N = 210)**  ***M (SD)*** | **Good Outcome Group (N = 102)**  ***M (SD)*** | **Poor Language Outcome Group**  **(N = 34)**  ***M (SD)*** | **Poor Reading Fluency**  **Outcome Group**  **(N = 34)**  ***M (SD)*** | **Poor Reading Comprehension Outcome Group**  **(N = 38)**  ***M (SD)*** |
| --- | --- | --- | --- | --- | --- | --- |
| **4-year measures (risk factors)** | **Non-verbal** | -0.23 (1.16) | -0.04 (1.02) | -1.16 (1.07) | -0.73 (1.13) | -1.21 (1.20) |
|  | **Language** | -0.34 (1.16) | 0.05 (1.05) | -1.36 (1.00) | -1.08 (1.06) | -1.30 (0.96) |
|  | **Phonological**  **Awareness** | -0.32 (1.03) | -0.22 (1.07) | -0.59 (0.83) | -0.68 (0.92) | -0.85 (0.81) |
|  | **Articulation** | -0.26 (1.15) | -0.02 (1.03) | -0.84 (1.16) | -1.00 (1.14) | -0.92 (1.19) |
|  | **Letter Knowledge** | 1.70 (1.17) | 1.79 (1.14) | 1.41 (1.26) | 1.35 (1.25) | 1.47 (1.22) |
|  | **Family History**  ***N (%)*** | 56 (26.7%) | 20 (19.6%) | 15 (44.1%) | 19 (55.9%) | 16 (42.1%) |
| **12-year measures (outcomes)** | **Language** | -0.09 (1.02) | 0.40 (0.74) | -1.60 (0.52) | -0.73 (1.02) | -0.90 (0.95) |
|  | **Reading**  **Fluency** | -0.08 (1.01) | 0.35 (0.78) | -0.67 (0.99) | -1.68 (0.61) | -0.77 (1.06) |
|  | **Reading**  **Comprehension** | -0.09 (1.04) | 0.43 (0.69) | -1.17 (0.81) | -0.90 (0.95) | -1.69 (0.55) |

Table S1: Profile of sample at age 4 and 12, overall and separately for outcome groups. Scores are z-scores for all variables except Family History and Letter Knowledge. (Letter Knowledge is a summed score from 0-4, indicating whether the child knows ‘no’ (0) ‘some’ (1) or ‘all’ (2) letter names, and ‘no’ (0) ‘some’ (1) or ‘all’ (2) letter sounds).

NB: Missing data for one or two (but not three) outcomes in some cases resulted in Language Outcome N = 174; Reading Fluency N = 205; Reading Comprehension N = 198.
