# Supplementary material for: Breadth versus depth: Cumulative risk model and continuous measure prediction of poor language and reading outcomes at 12
Source: Dev Sci. 2020 Jun 22;24(1):e12998. doi: 10.1111/desc.12998 (PMC11475567; doi:10.1111/desc.12998)
Supplement: Supplementary file 8 — Table S2 [file DESC-24-e12998-s003.docx]

Supplementary Online Materials 2

*Table S2. Indices of diagnostic validity for six CART risk prediction models*

| **Outcome variable** | **Risk factor measurement** | **Sensitivity** | **Specificity** | **Positive predictive value** | **Negative predictive value** |
| --- | --- | --- | --- | --- | --- |
| Oral language | Categorical | .44  (.27-.62) | .91  (.86-.95) | .56  (.39-.71) | .87  (.83-.90) |
| Oral Language | Continuous | .74  (.56-.87) | .96  (.91-.98) | .81  (.65-.90) | .94  (.89-.96) |
| Reading fluency | Categorical | .24  (.11-.41) | .98  (.95-1.0) | .73  (.43-.91) | .87  (.84-.96) |
| Reading fluency | Continuous | .62  (.44-.78) | .93  (.88-.96) | .64  (.49-.76) | .92  (.89-.95) |
| Reading comprehension | Categorical | .47  (.31-.64) | .96  (.91-.98) | .72  (.54-.85) | .88  (.85-.91) |
| Reading comprehension | Continuous | .42  (.26-.59) | .98  (.95-1.0) | .84  (.62-.95) | .88  (.84-.90) |

Figure legends for Figures S1a, S1b, S2a, S2b, S3a, S3b

Figure S1a: Classification and Regression Tree (CART): Poor Language Outcome at 12, based on categorical predictors at age 4.

Figure S1b: Classification and Regression Tree (CART): Poor Language Outcome at 12, based on continuous predictors at age 4.

Figure S2a: Classification and Regression Tree (CART): Poor Reading Fluency Outcome at 12, based on categorical predictors at age 4.

Figure S2b: Classification and Regression Tree (CART): Poor Reading Fluency Outcome at 12, based on continuous predictors at age 4.

Figure S3a: Classification and Regression Tree (CART): Poor Reading Comprehension Outcome at 12, based on categorical predictors at age 4.

Figure S3b: Classification and Regression Tree (CART): Poor Reading Comprehension Outcome at 12, based on continuous predictors at age 4.
